# Berberine Protects against Hepatocellular Carcinoma Progression by Regulating Intrahepatic T Cell Heterogeneity

**DOI:** 10.1002/advs.202405182

**Published:** 2024-08-13

**Authors:** Jiaxiang Hu, Qingmiao Shi, Chen Xue, Qingqing Wang

**Affiliations:** ^1^ Institute of Immunology Zhejiang University School of Medicine Hangzhou 310058 China; ^2^ Liangzhu Laboratory Zhejiang University Medical Center Hangzhou 311121 China; ^3^ State Key Laboratory for Diagnosis and Treatment of Infectious Diseases The First Affiliated Hospital, Zhejiang University School of Medicine Hangzhou 310003 China

**Keywords:** Berberine, CyTOF, Hepatocellular carcinoma, scRNA‐seq, T cells

## Abstract

Accumulating evidence suggests that berberine (BBR) exhibits anti‐cancer effects in hepatocellular carcinoma (HCC). However, the mechanisms by which BBR regulates the immunological microenvironment in HCC has not been fully elucidated. In this study, a mouse model of orthotopic HCC is established and treated with varying doses of BBR. BBR showed effectiveness in reducing tumor burden in mice with HCC. Cytometry by time‐of‐flight depicted the alterations in the tumor immune landscape following BBR treatment, revealing the enhancement in the T lymphocytes effector function. In particular, BBR decreased the proportion of TCRb^hi^PD‐1^hi^CD69^+^CD27^+^ effector CD8^+^ T lymphocytes and increased the proportion of Ly6C^hi^TCRb^+^CD69^+^CD27^+^CD62L^+^ central memory CD8^+^ T lymphocytes. Single‐cell RNA sequencing further elucidates the effects of BBR on transcriptional profiles of liver immune cells and confirms the phenotypical heterogeneity of T lymphocytes in HCC immune microenvironment. Additionally, it is found that BBR potentially regulated the antitumor immunity in HCC by modulating the receptor‐ligand interaction among immune cells mediated by cytokines. In summary, the findings improve the understanding of BBR's impact on protecting against HCC, emphasizing BBR's role in regulating intrahepatic T cell heterogeneity. BBR has the potential to be a promising therapeutic strategy to hinder the advancement of HCC.

## Graphical abstract

BBR treatment effectively reduces tumor burden in mice with HCC, thereby protecting against HCC progression. Functionally, BBR potentially regulates the differentiation status and phenotypical heterogeneity of intrahepatic T lymphocytes in the immune microenvironment of HCC. Moreover, BBR may enhance antitumor immunity in HCC by modulating cell–cell communications mediated by receptor‐ligand interactions.

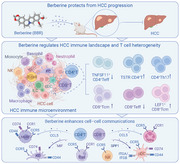

## 1 Introduction

Liver cancer ranks as the third leading cause of cancer‐related death, with hepatocellular carcinoma (HCC) being the most prevalent type of primary liver cancer.^[^ ^1^, ^2^ ^]^ Over the past decade, systemic therapies including immune checkpoint inhibitors (ICIs), tyrosine kinase inhibitors, and monoclonal antibodies have transformed the traditional treatment paradigm for HCC.^[^ ^3^ ^]^ Single‐agent ICIs are estimated to provide substantial clinical benefit for 15–20% of responders, but face the challenge of biomarkers failing to identify this population.^[^ ^4^, ^5^ ^]^ Therefore, it is necessary to explore novel therapeutic strategies to improve the landscape of HCC management.

Liver immune microenvironment plays a vital role in the tumorigenesis of HCC. For example, tumor‐infiltrating T cells and B cells have been observed interacting, leading to enhanced local immune activation and control of HCC progression.^[^ ^6^ ^]^ A study utilizing single‐cell RNA sequencing (scRNA‐seq) revealed that exhausted CD8^+^ T cells and infiltrating Tregs may undergo clonal expansion in the HCC microenvironment.^[^ ^7^ ^]^ Unlike the typical exhausted state observed in primary HCC, CD8^+^ T cells in early‐relapse HCC exhibit an innate‐like reduced cytotoxicity and clonal expansion, potentially explaining the compromised antitumor immune function and poor prognosis associated with HCC.^[^ ^8^ ^]^ Therefore, regulating the tumor immune microenvironment (TIME) may contribute to developing effective therapies for HCC.^[^ ^9^ ^]^

Berberine (BBR), an isoquinoline alkaloid derived from the roots, bark, and rhizomes of plants like *Coptis chinensis*, exhibits diverse pharmacological effects.^[^ ^10^ ^]^ A previous study demonstrated that BBR alleviates non‐alcoholic fatty liver disease by reducing gluconeogenesis and lipogenesis.^[^ ^11^ ^]^ Additionally, BBR was reported to inhibit HCC through activating PPARδ to trigger apoptotic death and promoting gut microbiota‐derived butyric acid production.^[^ ^12^ ^]^ In recent years, researchers have increasingly focused on the immunomodulatory properties of BBR. Liu and colleagues found that BBR exerted its antitumor effect in non‐small cell lung cancer by reducing PD‐L1 expression and enhancing tumor‐infiltrating T‐cells immunity.^[^ ^13^ ^]^ Another study revealed that BBR promoted the transformation of immunosuppressive M2 macrophages into tumoricidal M1 macrophages, restoring the antitumor cytotoxicity of T cells.^[^ ^14^ ^]^ However, the precise mechanism of BBR in regulating the immune response in HCC remains undiscovered.

In this study, we investigated the effects of BBR on HCC and elucidated its immunoregulatory role in the TIME of HCC. We found that BBR could notably decrease tumor weight and volume in a mouse model of orthotopic HCC. Utilizing cytometry by time‐of‐flight (CyTOF), we identified immune cell landscape in HCC. Additionally, the single‐cell transcriptional profile of CD45^+^ immune cells was constructed through scRNA‐seq. BBR administration led to an increase in central memory CD8^+^ T (Tcm) cells and a decrease in effector T (Teff) cells expressing high levels of inhibitory receptors, thereby alleviating T cell exhaustion. Cytokine detection revealed the regulatory effect of BBR on T cell function. Our findings comprehensively delineate the impact of BBR on immune cell landscape of HCC and suggest the potential efficacy of BBR as an immune modulatory strategy in vivo.

## 2 Results

### 2.1 BBR Treatment Alleviates the Tumor Load in Mice with HCC

**Figure 1:**
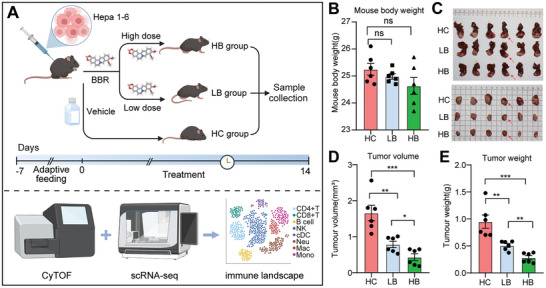
BBR treatment reduces tumor burden in HCC bearing mice. A) Experimental design. B) Mouse body weight of the tumor‐bearing mice from HC, LB, and HB group. C) Gross examination of liver tumors of the HC, LB, and HB group. D) Tumor volume of the tumor‐bearing mice from HC, LB, and HB group. E) Tumor weight of the tumor‐bearing mice from HC, LB, and HB group. ns, non‐significant; ^∗^ *p* < 0.05; ^∗∗^ *p* < 0.01; ^∗∗∗^ *p* < 0.001.

To investigate the potential of BBR in the treatment of HCC, we established a mouse model of orthotopic HCC. Following one week of adaptive feeding, mice were injected with the Hepa 1–6 cells into the left lobe of liver and subsequently received intraperitoneal BBR administration. The mice were categorized into three groups based on BBR dosage: HC group (vehicle administration), LB group (low‐dose BBR, 10 mg kg^−1^), and HB group (high‐dose BBR, 30 mg kg^−1^). BBR was administered every 2 days for a total of 14 days. Upon euthanasia, mouse livers were harvested and tumor tissues were excised for further analysis (**Figure** **1**A). The results revealed no significant differences in mouse body weight among the three groups (Figure 1B). However, mice treated with BBR exhibited obviously diminished tumor volume (Figure 1C). In particular, compared to the LB group, the high‐dose BBR treatment displayed a more significant reduction in tumor volume and weight (p < 0.05), indicating a dose‐dependent trend of BBR in alleviating the burden of HCC in mice (Figure 1D,E). Taken together, BBR demonstrated efficacy in reducing tumor burden in mice model, suggesting it's potential function in protecting from HCC progression.

### 2.2 CyTOF Depicted the Alterations in the Tumor Immune Landscape Following BBR Treatment

**Figure 2:**
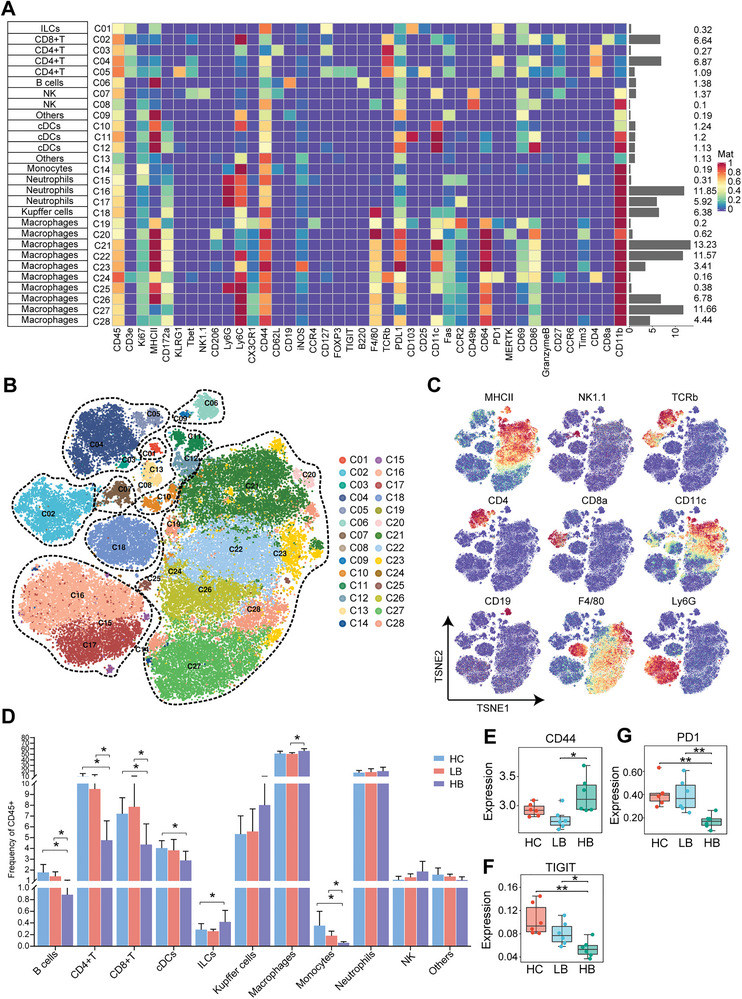
CyTOF depicted the alterations in the tumor immune landscape following BBR treatment. A) Heatmaps depicting the expression of 42 cell surface markers across 28 immune cell subsets. B) t‐SNE plot visualizes the 28 immune cell subsets. C) t‐SNE plot presents the expression of characteristic surface markers across immune cell subsets. D) Differential analysis in frequency of eleven immune cell types after treatment with BBR. E–G) The marker expression levels of (E) CD44, (F) TIGIT, and (G) PD‐1. ^∗^ *p* < 0.05; ^∗∗^ *p* < 0.01.

The tumor tissues collected from the three groups of the murine HCC model were processed into single‐cell suspensions, followed by CyTOF procedure to analyze alterations in the immune landscape of the HCC tumor microenvironment (TME) in response to BBR treatment. Subsequent to initial data processing, we conducted a cluster analysis of the immune cells using the Phenograph algorithm, resulting in the classification of these immune cells into 28 clusters based on classical markers (Table S1, Supporting Information). The 28 clusters were further categorized into 10 primary cell types, namely innate lymphoid cells (ILCs), CD8^+^ T lymphocytes, CD4^+^ T lymphocytes, B lymphocytes, natural killer (NK) cells, classical dendritic cells (cDCs), monocytes, neutrophils, Kupffer cells, and macrophages (**Figure** **2**A). In addition, we used t‐SNE plots to illustrate the dimensionality reduction clustering outcomes of the 28 immune cells clusters (Figure 2B) and depict the expression of canonical cell surface markers in various immune cells (Figure 2C).

A comparison of immune cell subsets between the HC, LB, and HB groups revealed notable alterations in the HCC immune landscape following BBR treatment. The most significant changes occurred in the T lymphocyte and macrophage. Compared to HC group, the proportion of CD4^+^ and CD8^+^ T lymphocytes decreased in the high‐dose group, while such changes were not significant in the low‐dose group. Macrophages and T lymphocytes exhibited opposite trends, as the proportion of macrophages in the high‐dose group increased compared to the low‐dose group (Figure 2D; Figure S1, Supporting Information). In mice treated with BBR, in addition to changes in their proportions, T lymphocyte subsets showed notable changes in functional characteristics. Compared with the control group, the high‐dose group exhibited a significant increase in the expression abundance of CD44, a marker that represents the functional activation of T lymphocyte (Figure 2E), indicating enhanced T lymphocyte antitumor activity. Moreover, markers of T lymphocyte exhaustion, TIGIT, and PD‐1, exhibited decreased expression after BBR treatment, indicating attenuation of T cell dysfunction and robust activation (Figure 2F,G).

### 2.3 BBR Treatment Regulated the Heterogeneity of T Lymphocytes in Mice with HCC

**Figure 3:**
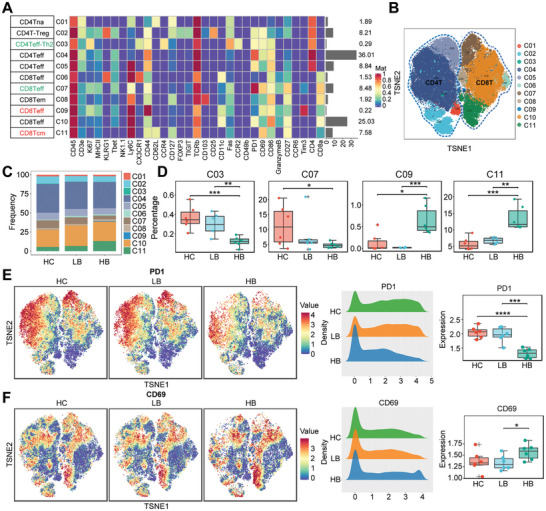
BBR treatment regulated the heterogeneity of T lymphocytes in mice with HCC. A) T lymphocyte subsets pedigree markers heatmap. B) The t‐SNE plot of T lymphocyte subsets. C) The proportional distribution of T lymphocyte subsets. D) Variations in the proportion of C03, C07, C09, and C11 subsets among HC, LB, and HB group. E) The expression of functional marker PD‐1 among the three groups. F) The expression of functional marker CD69 among the three groups. ^∗^ *p* < 0.05; ^∗∗^ *p* < 0.01; ^∗∗∗^ *p* < 0.001.

T lymphocytes play pivotal roles in targeting and killing specific cells, regulating cytokine secretion and immune processes.^[^ ^15^ ^]^ In this study, a decrease in the proportion of T lymphocytes was observed in HCC‐bearing mice treated with a high dose of BBR. Therefore, we performed a stepwise cluster analysis on the of T lymphocytes, aiming to explore the heterogeneity of T lymphocyte subsets following treatment with BBR. T lymphocytes were subcategorized into 11 clusters based on canonical T cell markers (**Figure** **3**A). T‐SNE plots were generated to display the distribution and quantity of each cell cluster (Figure 3B). Following BBR treatment, we visually observed alterations in the immune landscape through t‐SNE plots (Figure S2A, Supporting Information). Statistical analysis revealed significant changes in the proportions of C03, C07, C09, and C11 subsets post‐BBR treatment (Figure 3C,D). Notably, C07, characterized by TCRb^hi^PD‐1^hi^CD69^+^CD27^+^, represented an CD8^+^ Teff lymphocytes with high PD‐1 expression (Table S2, Supporting Information). Conversely, C11, featured by Ly6C^hi^TCRb^+^CD69^+^CD27^+^CD62L^+^, represented a subset of CD8^+^ Tcm lymphocytes with potential tumor‐killing characteristics. After treatment, the TCRb^hi^PD‐1^hi^CD69^+^CD27^+^ CD8^+^ Teff decreased in proportion in the HB group, while the proportion of Ly6C^hi^TCRb^+^CD69^+^CD27^+^CD62L^+^ CD8^+^ Tcm increased, underscoring the enhanced ability of the immune system to effectively eradicate tumor cells.

A comprehensive analysis of functional markers within T lymphocytes revealed decreased expression of markers indicative of functional exhaustion, such as PD‐1, TIGIT, and FOXP3 (Figure 3E; Figure S2B,C, Supporting Information). In contrast, the expression of markers suggestive of functional activation, such as CD69 and CD25, was increased (Figure 3F; Figure S2D, Supporting Information). These findings collectively indicate that BBR treatment regulated the heterogeneity of T lymphocyte in mice with HCC, leading to an overall improvement in the effector function of T lymphocytes. Specifically, BBR therapy counteracted T lymphocyte exhaustion by diminishing the proportion of CD8^+^Teff lymphocytes characterized by high expression of inhibitory receptors, while increasing the proportion of CD8^+^ Tcm lymphocytes.

### 2.4 scRNA‐seq Elucidated the Global Liver Immune Profiles Respond to BBR Treatment

**Figure 4:**
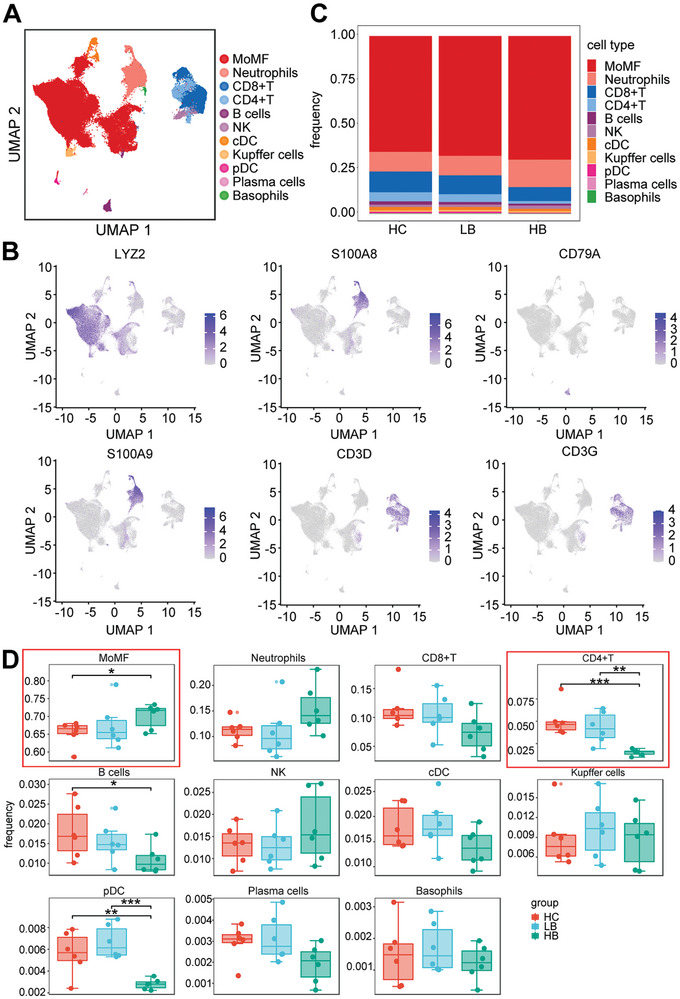
scRNA‐seq elucidated the global liver immune profiles respond to BBR treatment. A) UMAP plot of the cluster analysis of all samples, with each color denoting a distinct cell type. B) Expression profiles of specific marker genes for each cell type in UMAP. C) Proportion of immune cell types in the HC, LB, and HB groups. D) Alterations in the proportions of distinct cell types in the HC, LB, and HB groups. ^∗^ *p* < 0.05; ^∗∗^ *p* < 0.01; ^∗∗∗^ *p* < 0.001.

To further elucidate the effects of BBR treatment on liver immune profiles of mice with HCC, we collected mouse liver cancer tissue samples (n = 6:6:6) from HC group, LB group, and HB group. Single‐cell suspension was prepared using the method established in the preceding phase of the experiment. Subsequently, scRNA‐seq was performed to obtain single‐cell transcriptional profiles. An unsupervised cluster analysis of the scRNA‐seq data annotated eleven distinct immune cell types based on established marker genes, including monocyte‐derived macrophages (MoMFs), cDCs, neutrophils, basophils, Kupffer cells, CD4^+^ T cells, CD8^+^ T cells, NK cells, plasma cells, B cells, and plasmacytoid dendritic cells (pDCs) (**Figure** **4**A). Myeloid cells exhibited specific expression of LYZ2 genes, while neutrophils specifically expressed S100A9 and S100A8 genes. B cells demonstrated specific expression of CD79a genes, whereas T/NK cells specifically expressed CD3g and CD3d genes (Figure 4B). It is noteworthy that MoMFs, T/NK cells, and neutrophils constituted the predominant cell types across the three groups (Figure 4C). Furthermore, various other immune cell populations were present in the liver of HCC mice, albeit in smaller proportions, indicating heterogeneity within TIME. Compared with that in the HC group, the proportion of MoMFs in the HB group exhibited an increasing trend (Figure 4D). Conversely, the proportion of CD4^+^ T cells was decreased in the HB group. After BBR treatment, the difference in the proportion of immune cells mainly centered on T cells and MoMFs, and the overall trend was consistent with the CyTOF results.

### 2.5 scRNA‐seq Confirmed the Phenotypical Heterogeneity of T Lymphocytes in the Immune Microenvironment of HCC

**Figure 5:**
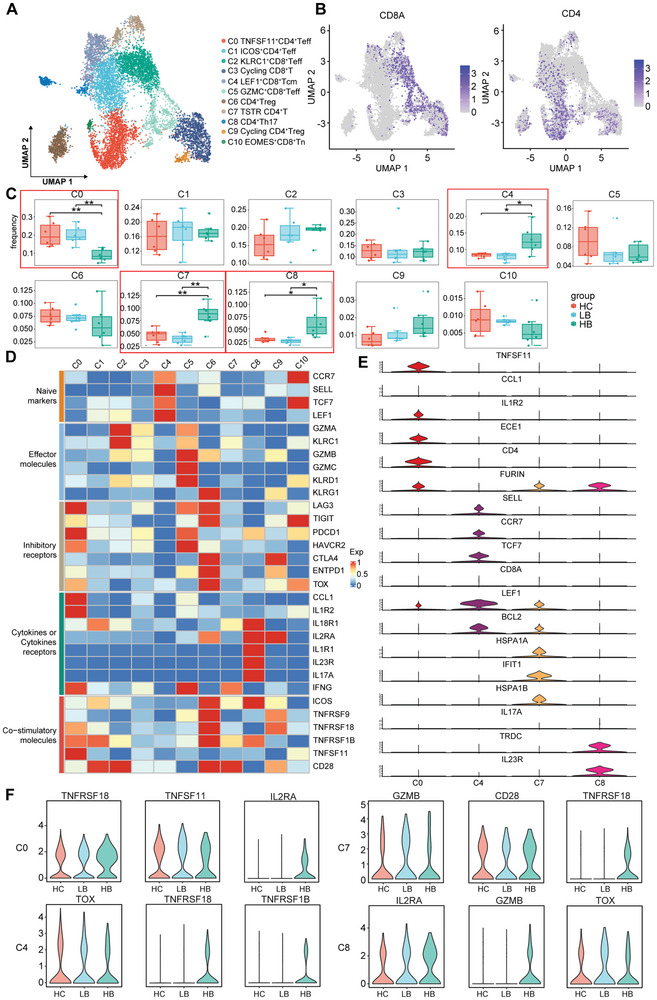
scRNA‐seq confirmed the phenotypical heterogeneity of T lymphocytes in the immune microenvironment of HCC. A) UMAP plot of T lymphocyte clusters in all samples. B) UMAP plot of marker CD4 and CD8a. C) Differences in the proportion of distinct T lymphocyte clusters in the HC, LB, and HB groups. D) Heatmap illustrating the characteristic genes across 11 clusters of T lymphocytes. E) Violin plot displaying characteristic genes in C0, C4, C7, and C8 clusters. F) Violin plot displaying the expression of characteristic genes in C0, C4, C7, and C8 clusters, respectively. ^∗^ *p* < 0.05; ^∗∗^ *p* < 0.01.

To discern phenotypical heterogeneity of T cells, we conducted unsupervised cluster analysis on scRNA‐seq data comprising 8626 T cells from three sample sets (**Figure** **5**A,B). According to the characteristic genes, 11 distinct T lymphocyte clusters were identified: C0 (TNFSF11^+^CD4^+^Teff), C1 (ICOS^+^CD4^+^Teff), C2 (KLRC1^+^CD8^+^Teff), C3 (Cycling CD8^+^T), C4 (LEF1^+^CD8^+^Tcm), C5 (GZMC^+^CD8^+^Teff), C6 (CD4^+^Treg), C7 [T‐cell stress response state (TSTR) CD4^+^T], C8 (CD4^+^Th17), C9 (Cycling CD4^+^Treg), and C10 (EOMES^+^CD8^+^Tn) (Table S3, Supporting Information). Compared to the HC group, high‐dose BBR treatment resulted in reduced frequency of immunosuppressive TNFSF11^+^CD4^+^ Teff cell expressing IL1R2 and increased frequency of LEF1^+^CD8^+^ Tcm cell potentially undergoing differentiation (Figure 5C). Moreover, the TSTR CD4^+^ T cell and pro‐inflammatory CD4^+^ Th17 cell expressing IL23R were also observed to be significantly elevated in the HB group.

To further investigate the functional alterations within the T lymphocytes, we analyzed gene expression levels concerning naive markers, effector molecules, inhibitory receptors, cytokine or cytokine receptors, and co‐stimulatory molecules (Figure 5D). The results showed that the inhibitory receptors gene LAG3 and PDCD1 were highly expressed in TNFSF11^+^CD4^+^ Teff cell. Characteristic genes indicating T cell differentiation potential, such as LEF1, exhibited robust expression in LEF1^+^CD8^+^ Tcm cells. The co‐stimulatory molecule CD28 was significantly expressed on TSTR CD4^+^ T cells, while the gene of cytokines or cytokine receptors were obviously expressed on CD4^+^ Th17 cells. Next, our investigation revealed significant differences in the gene expression profiles within these four clusters (Figure 5E). Specifically, expressions of TNFSF11, IL1R2, ECE1, CD4, and FURIN were upregulated in TNFSF11^+^CD4^+^ Teff cells, while expressions of SELL, CCR7, TCF7, LEF1, and BCL2 were elevated in LEF1^+^CD8^+^ Tcm cells. TSTR CD4^+^ T cells significantly expressed FURIN, LEF1, BCL2, HSPA1a, HSPA1b, and IFIT1, whereas CD4^+^ Th17 cell showed significant expression of FURIN, TRDC, and IL23R.

In addition, we investigated the effects of BBR treatment on the expression levels of characteristic genes in C0, C4, C7, and C8 (Figure 5F). IL2RA, a characteristic gene of TNFSF11^+^CD4^+^ Teff cells, was significantly upregulated following treatment with a high dose of BBR. TNFRSF18, which can significantly reverse CD4^+^ Treg cell‐mediated immunosuppression as a T cell co‐stimulatory signal, was highly expressed in LEF1^+^CD8^+^ Tcm and TSTR CD4^+^ T cells in HB group. GZMB, which can inhibit aberrant IL‐17 production in CD4^+^ T cells, thereby impeding angiogenesis and subsequent tumor development, was upregulated in CD4^+^ Th17 cells in HB group. These findings suggest that T cells exhibit heterogeneity in the immune microenvironment of HCC, and BBR treatment regulate antitumor immunity by affecting the expression of characteristic genes in T cells.

### 2.6 BBR Treatment Potentially Regulated the Level of Cytokines Secreted by Heterogeneous T Cells

**Figure 6:**
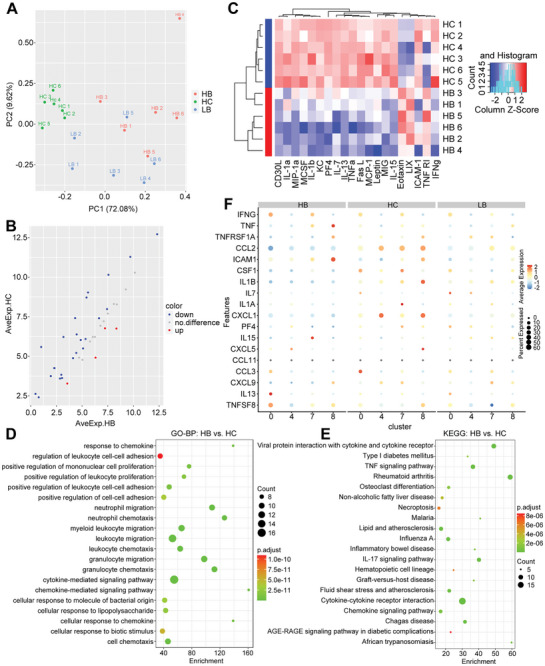
BBR treatment potentially regulates the immune microenvironment by modulating cytokine secretion. A) PCA plot of cytokines in the HB, LB, and HC groups. B) Differential expression of cytokines between the HB and HC groups. C) Heatmap illustrating the differences in cytokines between the HB and HC groups. D) Dot plot depicting the alterations of cytokines among the HB, LB, and HC groups. E) GO‐BP enrichment analysis. F) KEGG pathway enrichment analysis.

To further explore the potential regulatory effect of BBR on the murine HCC immune microenvironment, we quantitatively analyzed 40 cytokines in mouse serum (Table S4, Supporting Information). The principal component analysis (PCA) plot indicated clear distinctions among samples from HC, LB, and HB groups (**Figure** **6**A). Next, we analyzed the differential expression of cytokines between the HC and HB groups (Figure 6B). Cluster heatmap showed that most cytokines were significantly down‐regulated by high‐dose BBR treatment, such as IL‐1α, IL‐1β, IL‐7, IL‐13, IL‐15, TNF‐α, monocyte chemotactic protein 1 (MCP‐1), and leptin (Figure 6C). GO and KEGG enrichment analysis revealed that the differentially expressed cytokines proteins primarily involved in biological processes related to cytokine and chemokine‐mediated signaling pathways (Figure 6D,E).

Next, we investigated the relationship between heterogeneous T cells and their expression of cytokines gene based on scRNA‐seq data (Figure 6F). The results showed that high‐dose BBR administration resulted in downregulated expression of C‐C chemokine ligand (CCL) 2, colony‐stimulating factor‐1 (CSF‐1), IL‐1β, and C‐X‐C motif chemokine ligand (CXCL) 1. Conversely, the TNFSF11^+^CD4^+^ Teff cells exhibited upregulated expression of Interferon‐γ and IL‐13, while CCL3 expression was downregulated. IL‐15, known for inducing T cell activation and maintain T cell effector function, was predominantly expressed by TSTR CD4^+^ T cells in the HB group. Additionally, CD4^+^ Th17 in the HB group exhibited substantial secretion and expression of TNF. This indicates that the secretion of cytokines is linked to heterogeneous T cells and is influenced by high‐doses BBR.

### 2.7 BBR Enhanced Cell–Cell Interactions and Communications of Immune Cells in HCC

**Figure 7:**
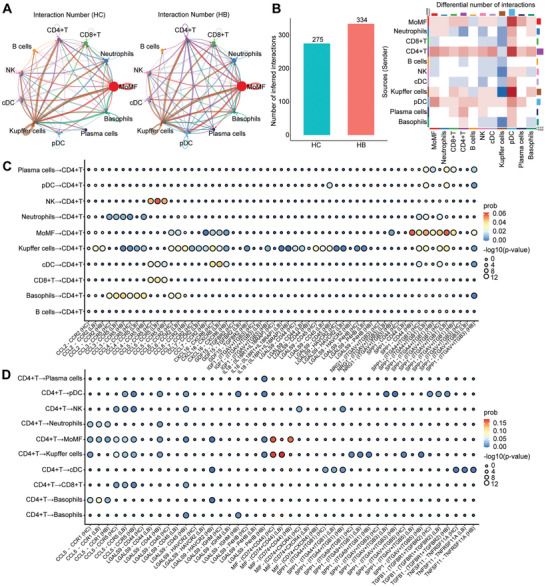
BBR enhances cell–cell interactions and communications of immune cells in HCC. A) The number of intercellular interactions in the HC and HB groups, respectively. B) Bar plot presents the interaction number of the HC and HB groups, the heatmap represents the differential number of interactions comparing HB groups to HC group. C) Bubble map presenting the comparison of significant ligand–receptor pairs in the HB, LB, and HC groups, when CD4^+^ T cell as receptor cells. D) Bubble map presenting the comparison of significant ligand–receptor pairs in the HC, LB, and HB groups, when CD4^+^ T cell as ligand cells.

CellChat was employed to determine the ligand‐receptor interactions and communication networks among various immune cell types (**Figure** **7**A; Figure S3A, Supporting Information). Compared to the HC group, we observed an overall increase in the number of intercellular interactions after high‐dose BBR treatment (Figure 7B). Ligand‐receptor analysis elucidated that high‐dose BBR predominantly regulated the signal transduction from liver immune cell to CD4^+^ T cells through chemokines ligand‐receptor pairs, such as CCL3–CCR5, CCL5–CCR5, CXCL16–CXCR6, as well as SPP1–(ITGA+ITGB) ligand/receptor pairs, such as SPP1–(ITGA4+ITGB1), SPP1–(ITGAV+ITGB1) (Figure 7C). In addition, BBR mainly mediates intercellular communication from CD4^+^ T cells to immune cells such as MoMF and Kupffer cell through ligand‐receptor pairs including CCL5–CCR1, CCL5–CCR5, macrophage migration inhibitory factor (MIF)–(CD74+CD44), LGALS9– CD44, LGALS9–CD45, LGALS9–HAVCR2, and SPP1–(ITGA+ITGB) (Figure 7D).

Additionally, high‐dose BBR regulated the interactions from liver cancer immune cells to CD8^+^ T cells via ligand‐receptor pairs such as CCL3–CCR5, CCL5–CCR5, CXCL6–CXCR6, and SPP1–(ITGA4+ITGB1) (Figure S3B, Supporting Information). BBR mainly mediates the communication from CD8^+^ T cells to other immune cells through ligand‐receptor pairs such as CCL5–CCR1, CCL5–CCR5, and SPP1–(ITGA+ITGB) (Figure S3C, Supporting Information). Moreover, high‐dose BBR treatment can also induce specific signals in CD8^+ ^T cells, including TGFβ, IL1, and GDF (Figure S3D, Supporting Information). Collectively, the anti‐liver cancer effect of high‐dose BBR may be achieved via modulating the ligand‐receptor interactions among immune cells mediated by cytokines and chemokines, thereby regulating the liver immune microenvironment.

### 2.8 Pseudotime Analysis and Development Trajectories of T Lymphocyte

**Figure 8:**
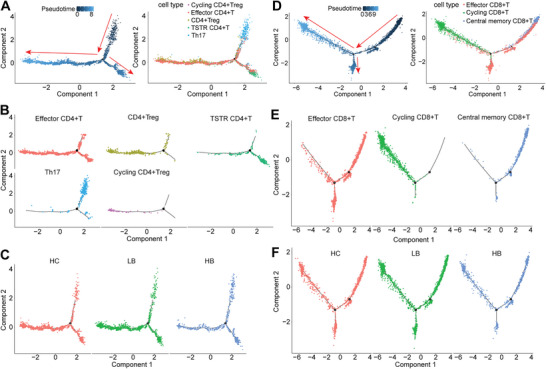
Pseudotime analysis and development trajectories of T lymphocyte clusters. A) Pseudotime trajectory of CD4^+^ T cells. B) Mapping of each CD4^+^ T cell cluster on a locus diagram. C) Cell trajectories of CD4^+^ T cells in the HC, LB, and HB groups. D) Pseudotime trajectory of CD8^+^ T cells. E) Mapping of each CD8^+^ T cell cluster on a trajectory diagram. F) Cell trajectories of CD8^+^ T cells in the HC, LB, and HB groups.

The Monocle algorithm was utilized to obtain the pseudotime trajectory of CD4^+^ T cells (**Figure** **8**A). The trajectory map revealed that Th17 cells were mainly distributed at the beginning of the pseudotime path. Subsequently, Th17 cells developed into TSTR CD4^+^ T cells before differentiating into CD4^+^ Treg cells, with effector CD4^+^ T cells emerging early in the differentiation process (Figure 8B). A comparison of the pseudotime trajectories of CD4^+^ T cells between the HB and HC groups demonstrated that high‐dose BBR treatment might regulate the development and differentiation of CD4^+^ T cells (Figure 8C). To dissect the CD8^+^ T cell developmental trajectories that occurs during high‐dose BBR treatment, we arranged single CD8^+^T cells into pseudotime trajectory (Figure 8D). We found that central memory CD8^+^ T cells mainly existed in the initial developmental stages, indicative of their potential for differentiation and their role as reserve cells for antitumor immunity (Figure 8E). In addition, the distribution characteristics of effector CD8^+^ T cells on the pseudotime path suggested that they seem to be in the middle stage of development. Further analysis indicated that high‐dose BBR treatment may regulate the heterogeneity of CD8^+^ T cells by affecting their differentiation status (Figure 8F).

## 3 Discussion

In recent years, significant advancements have been made in the immunotherapy of HCC.^[^ ^16^ ^]^ Although ICIs have emerged as the first‐line treatment for patients with advanced HCC, only fewer than 30% of patients achieve complete or partial remission. Therefore, exploring effective strategies to enhance its efficacy has become a key element in the development of HCC immunotherapy. Studies have shown that natural products like saponins, polysaccharides, and flavonoids hold substantial potential in tumor immunotherapy, primarily due to their role in the remodeling of tumor‐immunosuppressive microenvironment.^[^ ^17^, ^18^ ^]^ In this study, we demonstrate that BBR exerts a tumor‐suppressive effect in mouse model of orthotopic HCC, possibly by modulating the distribution pattern and function of immune cells in TIME. Further analysis revealed a decline in exhausted T (Tex) cells and an augmentation in functionally activated T cells. To the best of our knowledge, this study is the first to elucidate that how BBR could regulate intrahepatic T cell heterogeneity to protect against HCC. Our findings may provide new mechanistic insights into the natural product BBR as a promising anti‐HCC candidate.

Exhaustion of tumor‐infiltrated lymphocytes, especially CD8^+^ T cells, is one of the physiological mechanisms for HCC to evade antitumor immune responses.^[^ ^19^ ^]^ Tex cells exhibit a loss of effector function, characterized by the expression of multiple immune checkpoints, such as cytotoxic T lymphocyte‐associated antigen 4 (CTLA4), PD‐1, T cell immunoglobulin and mucin‐domain containing‐3 (TIM3), TIGIT, and FOXP3.^[^ ^20^ ^]^ Blockading inhibitory receptors is expected to overcome T cell exhaustion, thus eliciting effective antitumor immunity.^[^ ^21^ ^]^ Our study found that BBR administration reduced the expression level of PD‐1, TIGIT, and FOXP3 on T cells, suggesting that BBR has the potential to restore T cell exhaustion. A recent clinical trial demonstrated that lenvatinib plus anti‐PD‐1 antibodies is effective and safety conversion therapy regimen for unresectable HCC, and the responders showed significant enrichment of CD8^+^ T cells.^[^ ^22^ ^]^ Banta et al. found that dual blockade of PD‐1 and TIGIT coinhibitory receptors on T cells restored costimulatory receptor CD226 signaling and optimized antitumor CD8^+^ T‐cell responses.^[^ ^23^ ^]^ FOXP3 is a key transcription factor of Treg and plays an important role in maintaining the immunosuppressive function of Treg.^[^ ^24^ ^]^ Depletion of FOXP3^+^ Tregs and PD‐1^+^ T cells can restore the antitumor capabilities of T cells and expand Teff cell populations.^[^ ^25^ ^]^ However, the mechanism by which BBR inhibits the expression of immune checkpoints is still not well understood and requires further exploration.

Our data revealed that BBR increased the percentage of Tcm with high CD69 and CD25 expression. Similarly, Li et al. demonstrated the notable effect of BBR in promoting the memory and effector differentiation of CD8^+^ T cells, mainly achieved through activating AMPK and STAT5.^[^ ^26^ ^]^ Another study showed that BBR antagonized the β‐catenin pathway by inhibiting β‐catenin translation and mTOR activity, thereby reducing the survival rate of HCC cells.^[^ ^27^ ^]^ During tuberculosis, Pahuja and colleagues found that BBR enrich lung‐resident memory T cells by modulating NOTCH3/AKT signaling pathway, leading to enhanced anti‐tubercular immunity.^[^ ^28^ ^]^ These studies suggest that BBR can effectively regulate different cellular signaling pathways, providing new insights into the immune regulatory mechanism of BBR. Moreover, it was reported that BBR exhibited non‐toxicity to normal hepatocytes and easily penetrated tissues and tumors.^[^ ^29^ ^]^ Therefore, BBR holds promise as an immunomodulator for HCC.

In this study, high‐dose BBR was found to protect against HCC progression, with decreased serum concentrations of IL‐1α, Leptin, MCP‐1, and TNF‐α. Similarly, BBR was reported to reduce the secretion of TNF‐α and IL‐10 by Th1 and Th17 cells in mice with autoimmune neuritis.^[^ ^30^ ^]^ It is well established that HCC arises from precancerous, oncogene‐induced transformation of senescent cells, which can secrete IL‐6, IL‐8, IL‐1α, Leptin/Leptin receptor, MCP‐1, etc. IL‐6 and TNF‐α have been reported to promote HCC through the interaction of signal transducer and activator of transcription protein 3 and nuclear factor kappa B. Besides, Qin et, al. found that BBR can hinder the function of antigen‐presenting cells by downregulating the co‐stimulatory molecules CD80 and CD86 and the cytokines IL‐6 and IL‐12.^[^ ^31^ ^]^ On the other hand, our findings revealed that the transcript profiles related to cytokines was significantly altered in T cells of the HB group, with downregulation of genes encoding CSF‐1 and IL‐1β. TNFSF11^+^CD4^+^ Teff cells with high expression of TNFSF11, which positively regulates IL‐1β, TNF‐α, and IL‐6, were significantly decreased in the HB group, leading to a decrease in the aforementioned cytokines. Numerous clinical studies have highlighted the pro‐tumorigenic effects of IL‐1α and IL‐1β, in which IL‐1β stimulates inflammatory mediators, promotes cellular invasion and immunosuppression.^[^ ^32^, ^33^ ^]^ Additionally, TNF exhibits diverse tumor‐promoting activities, such as stimulating cytokine cascade responses, fibrotic responses, and altering adhesion receptors.^[^ ^34^ ^]^ These findings imply that BBR treatment could alter the transcriptional profiling of immune cells and prevent HCC progression through influencing the secretion of cytokines.

This study is the first to characterize the HCC immune landscape and constructed the CD45^+^ single‐cell transcriptional profile in BBR‐treated mice. We elucidate the impacts of BBR on intrahepatic immune cells in mice with HCC, specifically heterogeneous T cells. However, this study still has some limitations. First, the exact molecular mechanisms through which BBR regulates T cell function remain elusive and require further explorations. Second, further studies are greatly needed to determine how BBR affects the receptor‐ligand interactions between T cell and different immune cells subpopulations. Third, it is necessary to investigate the combination of BBR and emerging immunotherapy for HCC to ensure favorable outcomes in clinical applications.

## 4 Conclusion

In conclusion, our findings indicated that BBR prevented the progression of HCC through regulating the heterogeneity of intrahepatic T lymphocytes, which involves increasing the proportion of central memory CD8^+^ T cells while decreasing effector CD8^+^ T cells to reverse T cell exhaustion. Additionally, BBR may inhibit HCC by regulating the secretion of cytokines by T lymphocytes and affecting their differentiation status. Our study offers new insights into the immunomodulatory mechanisms of BBR in HCC, indicating potential application prospects on HCC management.

## 5 Experimental Section

### Cell line and Cell Culture

The Hepa 1–6 liver cancer cell line was obtained from the American Type Culture Collection (ATCC, USA) and stored in cryopreservation at the Institute of Immunology, Zhejiang University. Following established protocols, the Hepa 1–6 cells were cultured in Dulbecco's Modified Eagle's Medium (Gibco, USA) supplemented with 10% fetal bovine serum (Gibco, USA) and 1% penicillin–streptomycin (Gibco, USA).^[^ ^35^ ^]^ Continuous cell culture was conducted in a sterile environment at 37 °C with a 5% CO_2_ concentration.

### Animals

Wild type C57BL/6 male mice, aged 8–10 weeks, were acquired from the Experimental Animal Center of Hangzhou Medical College and fed in the Laboratory Animal Center of Zhejiang University under specific pathogen‐free conditions. The mice were maintained on a 12‐h dark/light cycle at a temperature of 22–25°C with ad libitum access to food and water. All animal experiments complied with the guidelines for the Care and Use of Laboratory Animals of the National Research Council and were approved by the Zhejiang University Animal Ethics Committee (NO. ZJU20240129).

### BBR Solution

BBR with a purity of 98% was provided by Meilun Biology (Dalian, China), dissolved in dimethylsulfoxide (DMSO) (Aladdin, Shanghai, China) as a 10 mm stock solution, and kept at −20°C. For all intraperitoneally administration to mice, this stock solution was diluted in phosphate buffer saline (PBS) (Servicebio, Wuhan, China) to achieve the concentration of BBR working solution.

### Mouse Model of Orthotopic HCC

After a week of adaptive feeding, the wild type C57BL/6 mice were subject to construct a mouse model of orthotopic HCC.^[^ ^36^ ^]^ The detailed processes are as follows: the mice were anesthetized by intraperitoneal injections of 1% pentobarbital sodium solution at a dose of 50 mg kg^−1^. The mice were then placed on an experiment table in a supine position, and the abdominal cavity was exposed. 1 × 10^6^ Hepa1‐6 cells suspended in 10 µL 20% Corning Matrigel (Matrigel:PBS = 1:4) were injected into the left lobe of the liver. Subsequently, the liver was repositioned, and the peritoneum and skin were sutured. All surgical procedures were conducted meticulously to prevent additional injuries, and the wound was disinfected post‐operation. The mice were then placed in an incubator until they naturally regained consciousness.

Next, the HCC mouse models were randomly divided into three groups (6 mice in each group): HC group, LB group, and HB group. The HB group received a BBR working solution at a dosage of 30 mg kg^−1^, while the LB group received a BBR working solution at a dosage of 10 mg kg^−1^. The HC group was given vehicle only (0.01% DMSO in 99.9% PBS). The volume of administration was 100ul solution per 10 g of mice. Administration was carried out intraperitoneally every other day for a period of 14 days.

### Preparation of a Single‐Cell Suspension

Eighteen fresh HCC tissues were completely resected from the three groups of HCC mouse models (HC vs LB vs HB; n = 6 each) and preserved in tissue storage solution, then transported at a low temperature. Following washing with cell culture medium twice, the tissues were cut into 1 mm^3^ pieces. Then, the tissue pieces were placed in a C tube, into which a digestive enzyme mix (Mouse Tumor Dissociation Kit, Miltenyi Biotec, Germany) was added, and the tube was filled to a volume of 5 mL with cell culture medium. The tissues were dissociated by incubating at 37 °C for 1 h and were subsequently filtered through a 70‐µm cell strainer. The filtered tissues were subjected to centrifugation at 300 × *g* and a temperature of 4 °C to obtain the cells. Finally, the supernatant was aspirated, and the isolated single cells were resuspended for CyTOF and scRNA‐seq.

### CyTOF

Purified antibodies were labeled with metal tags using the MaxPAR Antibody Labeling Kit (Fluidigm, USA) and adjusted to the desired concentration prior to application (Table S5, Supporting Information). To exclude dead cells, the cells were washed with 1 × PBS and subsequently stained them with 100 µL of cisplatin solution (250 n _m_ ) on ice for 5 min. Subsequently, cells were incubated in an Fc receptor‐blocking solution before applying a mixture of surface antibodies to stain cells. Cells were then washed twice with fluorescence‐activated cell sorting (FACS) buffer (1 × PBS containing 0.5% bovine serum albumin, BD Bioscience, USA) and fixed overnight in 200 µL of intercalation solution (Maxpar Fix and Perm Buffer containing 250 nm 191/193Ir, Fluidigm, USA). Following fixation, cells were washed with FACS buffer and then with perm buffer (eBioscience, USA) and stained with an intracellular antibody cocktail for 30 min on ice. Subsequently, cells were washed and resuspended in deionized water, mixed with 20% EQ4 Element beads (Fluidigm, USA). The data were obtained on a Helios mass cytometer (Fluidigm, USA), then normalized and gated in FlowJo (version 10.1, BD, USA) or uploaded to Cytobank (Santa Clara, CA) for subsequent analysis. t‐Distributed stochastic neighbor embedding (t‐SNE) algorithm was utilized to visualize high‐dimensional data.^[^ ^37^ ^]^

### ScRNA‐seq

A single‐cell suspension containing over 90% living cells was acquired, with the cell concentration adjusted to 1000 cells/µL. According to the standard protocol, the single‐cell suspension was loaded onto the MobiNova−100 high‐throughput single‐cell controller device (MobiDrop, Zhejiang, China) to produce single‐cell gel beads in emulsions (GEMs).^[^ ^38^ ^]^ Then, the captured cell was lysed and the released mRNAs were reverse‐transcribed into complementary DNA (cDNA) with barcode. The GEMs were then disrupted to mix cDNAs from different cells. The resulting cDNAs were used as templates to amplify through polymerase chain reaction (PCR), and its quality was assessed using an Agilent 2100 (Agilent Technologies, USA). According to the manufacturer's instructions, scRNA‐seq libraries were prepared using MobiCube RNA‐seq Chip A Single Cell Kit v2.1 (MobiDrop, Zhejiang, China), which was then subjected to the MobiNova−100 single‐cell sequencing platform (MobiDrop, Zhejiang, China) with a 150 bp paired‐end reading strategy. After removing low‐quality reads and poly‐A tail and adapter sequences, the qualified raw data were processed to construct an expression profile matrix through Mobivision (v 1.1). The output gene expression matrices were analyzed through Seurat package (version 3.1.2) using the R software (version 4.0.3). After all cells were nonlinear dimensionally reduced via uniform manifold approximation and projection (UMAP) and projected into 2D space, cells were clustered together based on common characteristics.^[^ ^39^ ^]^ Individual cells in each cluster were annotated according to the gene expression of canonical markers for specific cell types. Marker genes exhibiting differential expression across these cell clusters were analyzed using the FindMarker function in the Seurat.

### Cell–Cell Communication Analysis

Cell**–**cell communication analysis was performed using the R package CellChat to investigate potential intercellular interaction.^[^ ^40^ ^]^ The “netVisual_circle” function was used to calculate the interactions number and strength between any two cell types. Visual representations illustrating total outgoing and incoming interaction probabilities were generated employing the function “netAnalysis_signalingRole_scatter”. The expression profiles of identified genes pertaining to ligand‐receptor pairs were presented in graphical format using the “Dotplot” function of Seurat.

### Pseudotime Analysis

The pseudotime trajectory was inferred using the Monocle (version 2) package.^[^ ^41^ ^]^ Highly variable gene expression profiles identified through scRNA‐seq were subjected to dimensionality reduction using the DDRTree algorithm for subsequent analysis. Subsequently, the function “orderCells” was employed to order each cell along pseudotime trajectory.

### Cytokine Quantification and Functional Enrichment Analysis

Following the provided instructions, 40 cytokines were identified from murine serum samples (HC vs LB vs HB; n = 6 each) utilizing the Quantibody Array Glass Chip (RayBiotech, USA). Raw data underwent preprocessing and normalization using RayBiotech software. Normalized data were analyzed employing moderated t‐statistics utilizing the limma data package from Bioconductor. Additionally, differentially expressed proteins were identified, with the cut‐off was set as to fold change > 1.2 or fold change < 0.83, and adjust p‐value < 0.05. Subsequent analysis results were visualized through scatter plots, volcano plots, and heatmaps.

### Statistical Analysis

GraphPad Prism (version 8.0) and R (version 4.0.3) were used for statistical analysis and graphing. The unpaired Student's t‐test were applied for comparisons between two groups. Data are the mean ± standard deviation. P value less than 0.05 were considered statistical significance. ^*^ *p* < 0.05, ^**^ *p* < 0.01 and ^***^ *p* < 0.001.

## Conflict of Interest

The authors declare no conflict of interest.

## Author Contributions

Q.W. and C.X. conceived and designed the study. J.H. and Q.S. performed the experiments and wrote the draft. J.H. participated in data acquisition and analysis. Q.W. and C.X. revised the manuscript. All authors read and approved the final manuscript.

## Supporting information

Supporting Information

## Data Availability

The data that support the findings of this study are available from the corresponding author upon reasonable request.
