# Supplementary material for: Berberine Protects against Hepatocellular Carcinoma Progression by Regulating Intrahepatic T Cell Heterogeneity
Source: Adv Sci (Weinh). 2024 Aug 13;11(39):2405182. doi: 10.1002/advs.202405182 (PMC11497054; doi:10.1002/advs.202405182)
Supplement: Supplementary file 1 — Supporting Information [file ADVS-11-2405182-s001.docx]

**Supplementary File**

**Berberine protects against hepatocellular carcinoma progression by regulating intrahepatic T cell heterogeneity**

Jiaxiang Hu^1,2^, Qingmiao Shi^3^, Chen Xue^3*^, Qingqing Wang^1,2*^

^1^ Institute of Immunology, Zhejiang University School of Medicine, Hangzhou, 310058, China

^2^ Liangzhu Laboratory, Zhejiang University Medical Center, Hangzhou, 311121, China

^3^ State Key Laboratory for Diagnosis and Treatment of Infectious Diseases, The First Affiliated Hospital, Zhejiang University School of Medicine, Hangzhou, 310003, China

***Corresponding author**:

**Qingqing Wang,** Institute of Immunology, Zhejiang University School of Medicine, No. 866 Yu Hang Tang Road, Hangzhou, Zhejiang 310058, China. E-mail: wqq@zju.edu.cn

**Chen Xue,** The First Affiliated Hospital, Zhejiang University School of Medicine, No. 79 Qingchun Road, Hangzhou, Zhejiang 310003, China. E-mail: zjuxuechen@zju.edu.cn

**The supplementary file includes:**

**Supplementary Figure 1-3**

**Supplementary Table 1-5
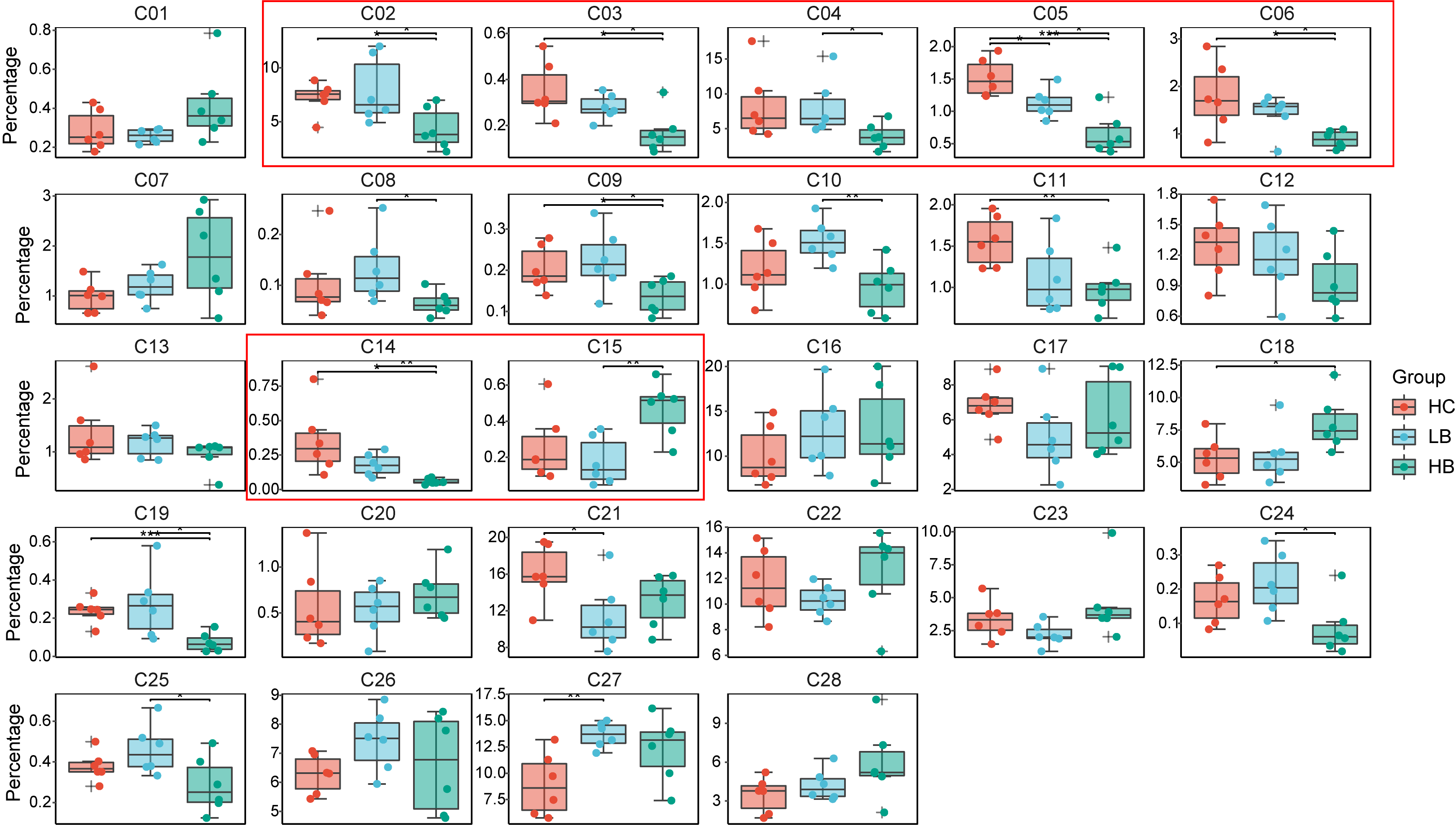
Figure. S1.** Box plot illustrating the alterations in proportions of 28 immune cell subsets across the HC, LB, and HB groups. ∗p < 0.05; ∗∗p < 0.01; ∗∗∗p < 0.001.

**
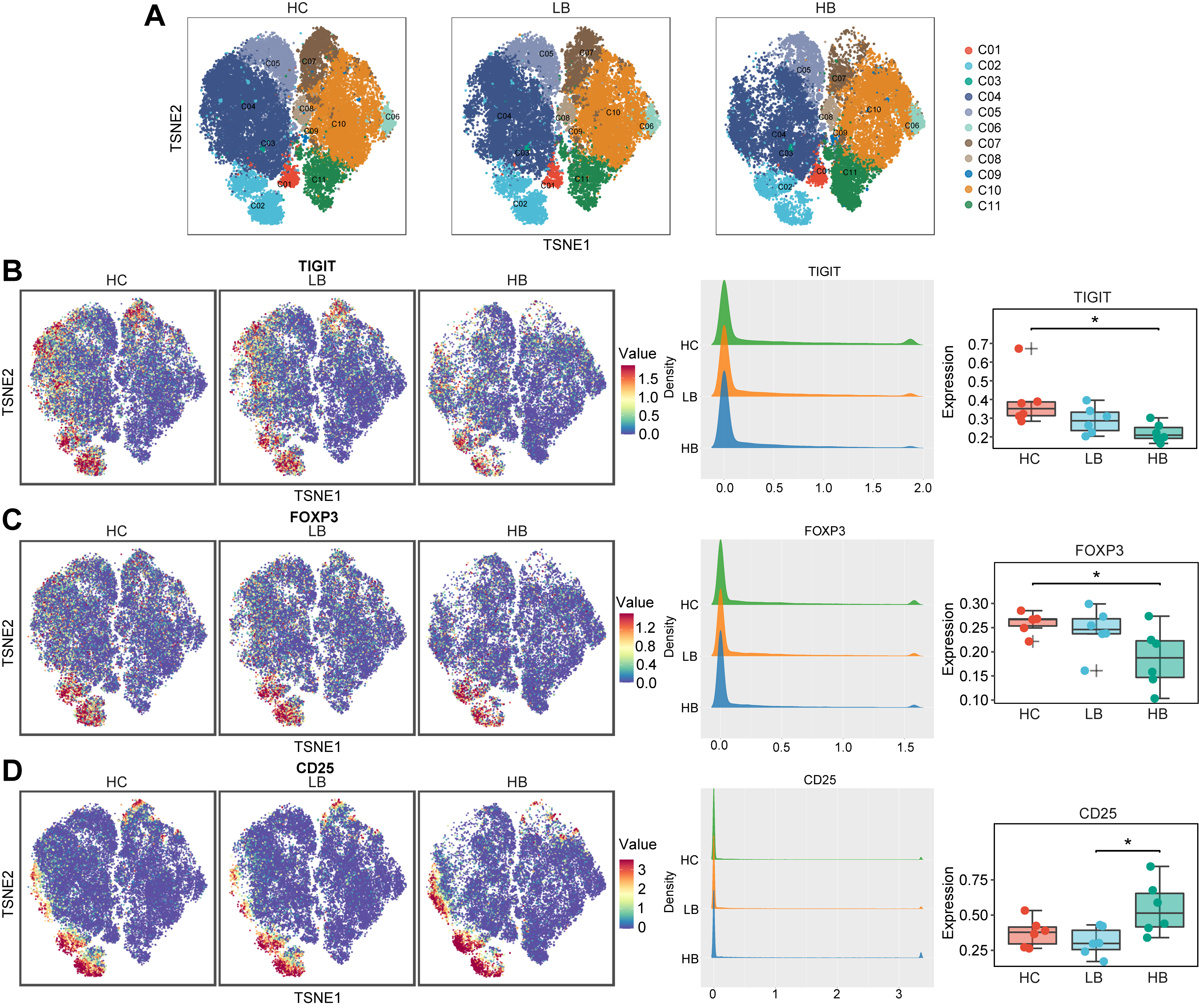
Figure. S2. A.** The t-SNE plots of T lymphocyte subsets in the HC, LB, and HB group, respectively. **B.** The expression of functional marker TIGIT among the three groups. **C.** The expression of functional marker FOXP3 among the three groups. **D.** The expression of functional marker CD25 among the three groups. ∗p < 0.05.

**
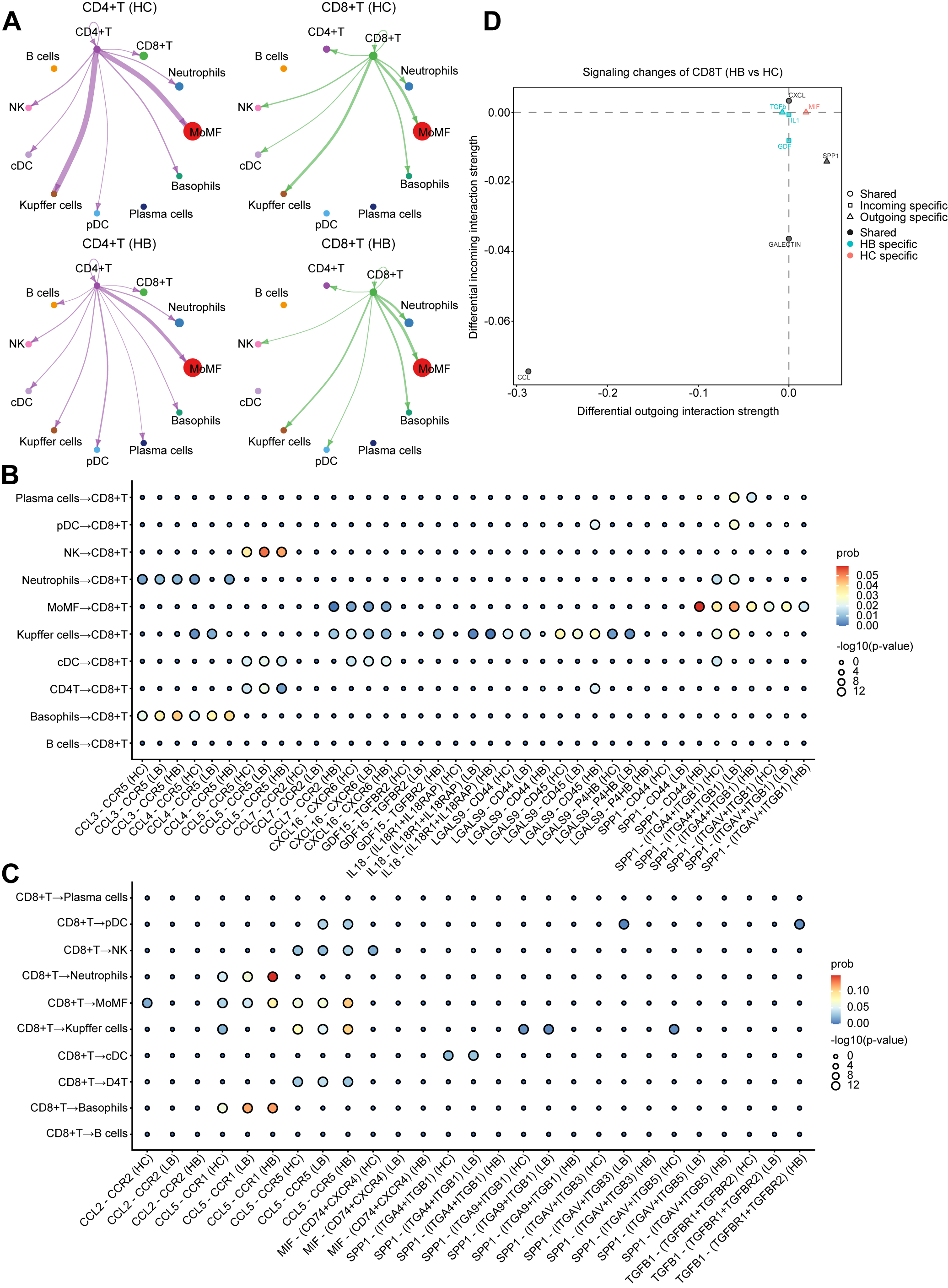
Figure. S3.** **A.** The quantity and intensity of intercellular interactions compared between the HC and HB groups. The section above represents the HC group, while the following section represents the HB group. **B.** Bubble map presenting the comparison of significant ligand–receptor pairs in the HB, LB, and HC groups, when CD8^+^ T cell as receptor cells. **C.** Bubble map presenting the comparison of significant ligand–receptor pairs in the HC, LB, and HB groups, when CD8^+^ T cell as ligand cells. Dot colors indicate the probability of communication between a specific ligand–receptor pair between a ligand cell and a receptor cell. **D.** CD8^+^ T cell signal variations in the HC group *versus* the HB group.**Supplementary Table 1. Markers expression of 28 immune cell clusters based on CyTOF analysis.**

| **Cluster** | **Cell types** | M**arker expression** |
| --- | --- | --- |
| C01 | ILC | CD45^+^, CD3e^+^, MHCII^+^, CD44^hi^, CD127^hi^, CD103^hi^, PD1^hi^, CD69^+^ |
| C02 | CD8^+^T | CD45^+^, Ly6C^hi^, TCRb^+^, PD1^+^, CD86^+^, CD27^+^, CD8a^+^ |
| C03 | CD4^+^T | CD45^+^, CD3e^+^, CD62L^+^, CD127^+^, TCRb^+^, PDL1^+^, CD4^+^ |
| C04 | CD4^+^T | CD45^+^, CD44^hi^, TCRb^+^, PD1^hi^, CD86^+^, CD4^+^ |
| C05 | CD4^+^T | CD45^+^, KLRG1^hi^, CD44^+^, CD127^+^, TCRb^+^, PDL1^+^, CD25^hi^, CD69^+^, CD4^+^ |
| C06 | B cells | CD45^+^, MHCII^hi^, CD19^+^, B220^+^ |
| C07 | NK | CD45^+^, NK1.1^+^, CD44^+^, PDL1^+^, CD11c^hi^, CD49b^hi^, CD69^+^, CD11b^hi^ |
| C08 | NK | CD45^+^, CD44^hi^, PDL1^hi^, CD49b^hi^, CD86^+^, CD11b^hi^ |
| C09 | Others | CD45^+^, Ki67^+^, MHCII^hi^, Ly6C^hi^, CD44^+^, PDL1^hi^, Fas^+^, CD69^+^, CD11b^+^ |
| C10 | cDCs | CD45^+^, MHCII^hi^, Ly6C^hi^, CD44^hi^, CD11c^hi^, CD11b^+^ |
| C11 | cDCs | CD45^+^, MHCII^hi^, CD44^hi^, PDL1^hi^, CD103^hi^, CD11c^hi^, CD86^+^, CD11b^hi^ |
| C12 | cDCs | MHCII^hi^ , CD172a^+^, CD44^hi^, PDL1^hi^, CD11c^hi^, CD86^hi^, CD11b^hi^ |
| C13 | Others | Ly6C^+^, CD44^hi^, PDL1^+^ |
| C14 | Monocytes | CD45^+^, Ki67^+^, Ly6C^hi^, CD44^hi^, CD11b^hi^ |
| C15 | Neutrophils | CD45^+^, Ki67^+^, Ly6G^+^, Ly6C^hi^, CD44^hi^, PDL1^+^, Tim3^hi^, CD11b^hi^ |
| C16 | Neutrophils | CD45^+^, Ly6G^+^, Ly6C^hi^, CD44^hi^, CD11b^hi^ |
| C17 | Neutrophils | CD45^+^, Ly6G^+^, Ly6C^hi^, CD44^hi^, CCR2^+^, CD11b^hi^ |
| C18 | Kupffer cells | CD45^+^, CD172a^+^ , Ly6C^+^, CD44^hi^, F4/80^hi^, PDL1^+^, CD11b^hi^ |
| C19 | Macrophages | CD45^+^, MHCII^hi^, Ly6C^+^, CX3CR1^+^, CD44^hi^, F4/80^+^, PDL1^+^, CD11c^+^, Fas^hi^, CCR2^hi^, CD64^hi^, CD69^+^, CD11b^hi^ |
| C20 | Macrophages | CD45^+^, Ki67^+^, MHCII^hi^, CD172a^+^, CD206^+^, CD44^hi^, F4/80^hi^, PDL1^hi^, CD11c^+^, CD64^hi^, MERTK^+^, CD86^hi^, Tim3^+^, CD11b^hi^ |
| C21 | Macrophages | CD45^+^, MHCII^hi^, CD172a^hi^, CD44^hi^, F4/80^hi^, PDL1^hi^, CD11c^hi^, CD64^hi^, CD86^hi^, CD11b^hi^ |
| C22 | Macrophages | CD45^+^, MHCII^hi^, CD172a^+^, Ly6C^hi^, CD44^hi^, F4/80^hi^, PDL1^hi^, CD11c^hi^, CD64^hi^, CD86^+^, CD11b^hi^ |
| C23 | Macrophages | CD45^+^, MHCII^hi^, CD172a^hi^, Ly6C^hi^, CD44^hi^, iNOS^hi^, F4/80^hi^, PDL1^hi^, CD11c^hi^, CD64^hi^, CD69^+^, CD86^+^, CD11b^hi^ |
| C24 | Macrophages | CD45^+^, MHCII^hi^, Ly6C^hi^, CD44^hi^, F4/80^+^, PDL1^hi^, CD11c^+^, CD64^hi^, PD1^+^, CD69^+^, CD86^hi^, CD4^+^, CD11b^hi^ |
| C25 | Macrophages | CD45^+^, MHCII^hi^, CD172a^hi^, Ly6G^+^, Ly6C^hi^, CD44^hi^, F4/80^hi^, PDL1^hi^, CD11c^+^, CD64^hi^, CD11b^hi^ |
| C26 | Macrophages | CD45^+^, MHCII^hi^, CD172a^+^, Ly6C^hi^, CD44^hi^, F4/80^hi^, PDL1^+^, CD64^hi^, CD86^+^, CD11b^hi^ |
| C27 | Macrophages | CD45^+^, Ly6C^hi^, CD44^hi^, F4/80^hi^, CD64^hi^, CD11b^hi^ |
| C28 | Macrophages | CD45^+^, CD172a^+^, Ly6C^hi^, CD44^hi^, F4/80^hi^, PDL1^hi^, CD11c^hi^, CD64^hi^, CD86^+^, CD11b^hi^ |

**Supplementary Table 2. Markers expression of 11 clusters of T lymphocytes based on CyTOF analysis.**

| **Cluster** | **Cell type** | **Marker Expression** |
| --- | --- | --- |
| C01 | CD4^+^Tna | TCRb^hi^, CD4^hi^ |
| C02 | CD4^+^T-Treg | KLRG1^hi^, CD44^+^, TCRb^hi^, CD25^+^, CD69^+^, CD4^hi^ |
| C03 | CD4^+^Teff | Ki67^+^, CD44^+^, CCR4^+^, TCRb^hi^, Fas^+^, PD1^+^, CD4^hi^ |
| C04 | CD4^+^Teff | CD44^hi^, TCRb^hi^, PD1^hi^, CD86^+^, CD4^hi^ |
| C05 | CD4^+^Teff | Ly6C^hi^, CD44^+^, TCRb^hi^, PD1^+^, CD86^+^, CD4^hi^ |
| C06 | CD8^+^Teff | KLRG1^hi^, Ly6C^hi^, CX3CR1^hi^, TCRb^hi^, CD11c^+^ |
| C07 | CD8^+^Teff | TCRb^hi^, PD1^hi^, CD69^+^, CD27^+^, CD8a^+^ |
| C08 | CD8^+^Tem | Ly6C^hi^, TCRb^hi^, CD103^hi^, CD86^+^, CD8a^+^ |
| C09 | CD8^+^Teff | Ly6C^hi^, CD44^hi^, TCRb^+^, CD86^+^, Tim3^hi^ |
| C10 | CD8^+^Teff | Ly6C^hi^, TCRb^hi^, CD86^+^, CD8a^+^ |
| C11 | CD8^+^Tcm | Ly6C^hi^, TCRb^+^, CD69^+^, CD27^+^ |

**Supplementary Table 3. The signature genes of 11 clusters of T lymphocytes based on** **scRNA-seq analysis.**

| **Cluster** | **Cell type** | **Signature gene** |
| --- | --- | --- |
| C0 | TNFSF11^+^CD4^+^Teff | TNFSF11, CCL1, IL1R2, ECE1, CD4, FURIN |
| C1 | ICOS^+^CD4^+^Teff | CD4, ICOS, IL18R1, FURIN |
| C2 | KLRC1^+^CD8^+^Teff | CD8a, GZMA, KLRC1, GZMB |
| C3 | Cycling CD8^+^T | MKi67, PLACF, TOP2a, BiRC5, CENPE |
| C4 | LEF1^+^CD8^+^Tcm | SELL, CCR7, TCF, CD8a, LEF1, BCL2 |
| C5 | GZMC^+^CD8^+^Teff | GZMC, GZMB, CD8a, GZMA, KLRD1 |
| C6 | CD4^+^Treg | IL2Ra, CTLA4, FOXP3, KLRG1, CD4 |
| C7 | TSTR CD4^+^T | HSPA1a, IFIT1, HSPA1b, CD4 |
| C8 | CD4^+^Th17 | IL17a, TRDC, IL23R, CD4, IL1R |
| C9 | Cycling CD4^+^Treg | MKi67, TOP2a, SMC2, FOXP3, IL2Ra, CD4 |
| C10 | EOMES^+^CD8^+^Tn | EomES, CCR7, TCF7, CD8 |

**Supplementary Table 4. Differences in the expression of 40 cytokines in mouse serum between HC and HB groups**

| **Cytokines** | **Average expression level** | | **LogFC** | **P value** | **Adjust P value** | **Regulation** |
| --- | --- | --- | --- | --- | --- | --- |
|  | **HC group** | **HB group** |  |  |  |  |
| CD30L | 2.628637 | 0.468747 | -2.15989 | 1.98E-06 | 4.21E-05 | down |
| IL-13 | 7.416806 | 4.324923 | -3.09188 | 2.10E-06 | 4.21E-05 | down |
| MIG | 9.034821 | 5.579229 | -3.45559 | 4.78E-06 | 6.38E-05 | down |
| MIP-1a | 5.589494 | 1.02171 | -4.56778 | 1.16E-05 | 0.000116 | down |
| Eotaxin | 4.918787 | 6.311526 | 1.39274 | 2.72E-05 | 0.000218 | up |
| Fas L | 6.384799 | 4.471056 | -1.91374 | 3.41E-05 | 0.000228 | down |
| LIX | 6.754251 | 8.337736 | 1.583484 | 0.000155 | 0.000888 | up |
| IL-15 | 10.15209 | 7.564368 | -2.58773 | 0.000558 | 0.002606 | down |
| PF4 | 11.28698 | 9.692812 | -1.59416 | 0.000586 | 0.002606 | down |
| KC | 4.252916 | 2.313946 | -1.93897 | 0.001174 | 0.004695 | down |
| IL-1a | 2.414505 | 0.774978 | -1.63953 | 0.002415 | 0.008049 | down |
| IL-7 | 6.223464 | 3.189499 | -3.03396 | 0.002278 | 0.008049 | down |
| IL-1b | 4.257443 | 2.283962 | -1.97348 | 0.003362 | 0.009605 | down |
| Leptin | 8.717217 | 5.428786 | -3.28843 | 0.003335 | 0.009605 | down |
| MCSF | 3.752814 | 1.953184 | -1.79963 | 0.003802 | 0.01014 | down |
| ICAM-1 | 7.985206 | 7.428598 | -0.55661 | 0.012053 | 0.030132 | down |
| MCP-1 | 6.034015 | 4.866276 | -1.16774 | 0.015025 | 0.033389 | down |
| TNF RI | 6.769058 | 7.230572 | 0.461514 | 0.014801 | 0.033389 | up |
| TNFa | 6.096798 | 5.340399 | -0.7564 | 0.016824 | 0.035419 | down |
| IFNg | 7.40677 | 6.650057 | -0.75671 | 0.02076 | 0.041519 | down |
| TIMP-1 | 12.71619 | 12.30682 | -0.40937 | 0.050554 | 0.096293 | down |
| GM-CSF | 5.216192 | 4.432501 | -0.78369 | 0.063004 | 0.109952 | down |
| IL-2 | 4.884863 | 4.119407 | -0.76546 | 0.063222 | 0.109952 | down |
| IL-4 | 3.628573 | 3.051706 | -0.57687 | 0.076474 | 0.127457 | down |
| IL-3 | 3.595982 | 2.850522 | -0.74546 | 0.081386 | 0.130217 | down |
| TARC | 3.835458 | 2.978538 | -0.85692 | 0.123499 | 0.189998 | down |
| MIP-1g | 8.307697 | 8.090518 | -0.21718 | 0.167101 | 0.247556 | down |
| Eotaxin-2 | 3.281769 | 3.56049 | 0.278721 | 0.195873 | 0.279818 | up |
| TCA-3 | 4.780168 | 5.037993 | 0.257825 | 0.250104 | 0.34497 | up |
| IL-12p70 | 5.496412 | 5.097237 | -0.39918 | 0.354972 | 0.473296 | down |
| IL-17 | 4.9214 | 4.704729 | -0.21667 | 0.40538 | 0.523071 | down |
| IL-6 | 7.704669 | 7.505941 | -0.19873 | 0.593256 | 0.719098 | down |
| RANTES | 5.941266 | 5.765561 | -0.1757 | 0.588312 | 0.719098 | down |
| IL-10 | 6.932203 | 6.779651 | -0.15255 | 0.612206 | 0.720242 | down |
| IL-5 | 7.219226 | 7.10192 | -0.11731 | 0.688476 | 0.786829 | down |
| BLC | 10.2862 | 10.13385 | -0.15235 | 0.784871 | 0.848509 | down |
| IL-21 | 6.223669 | 6.035893 | -0.18778 | 0.769754 | 0.848509 | down |
| G-CSF | 8.282486 | 8.382195 | 0.099708 | 0.850906 | 0.89569 | up |
| TNF RII | 9.231581 | 9.254518 | 0.022937 | 0.892711 | 0.915601 | up |
| MCP-5 | 6.226147 | 6.250334 | 0.024187 | 0.92078 | 0.92078 | up |

**Supplementary Table 5. Antibodies for CyTOF.**

| **No** | **Isotopes** | **Marker** | **Clone** | **Dilution** | **Provider** | **Catalog number** | **Intracellular** |
| --- | --- | --- | --- | --- | --- | --- | --- |
| 1 | 89Y | CD45 | 30-F11 | 400 | Biolegend | 103102 | / |
| 2 | 115ln | CD3ε | 145-2C11 | 50 | Biolegend | 100302 | / |
| 3 | 139La | Ki-67 | SolA15 | 400 | eB | 14-5698-82 | / |
| 4 | 141Pr | MHC II(I-A/I-E) | M5/114.15.2 | 400 | Biolegend | 107602 | intracellular |
| 5 | 142Nd | CD172a(SIRPα) | P84 | 50 | Biolegend | 144002 | / |
| 6 | 143Nd | KLRG1 | 2F1 | 100 | eB | 16-5893-82 | / |
| 7 | 144Nd | T-bet | 4B10 | 100 | Biolegend | 644802 | / |
| 8 | 145Nd | CD161(NK-1.1) | PK136 | 100 | Biolegend | 108702 | intracellular |
| 9 | 146Nd | CD206(MMR) | C068C2 | 100 | Biolegend | 141702 | / |
| 10 | 147Sm | Ly-6G | 1A8 | 200 | Biolegend | 127602 | intracellular |
| 11 | 148Nd | Ly-6C | HK1.4 | 400 | Biolegend | 128002 | / |
| 12 | 149Sm | CX3CR1 | SA011F11 | 400 | Biolegend | 149002 | / |
| 13 | 150Nd | CD44 | IM7 | 200 | Biolegend | 103002 | / |
| 14 | 151Eu | CD62L | MEL-14 | 800 | Biolegend | 104402 | / |
| 15 | 152Sm | CD19 | 6D5 | 400 | Biolegend | 115502 | / |
| 16 | 153Eu | iNOS | CXNFT | 50 | eB | 14-5920-82 | / |
| 17 | 154Sm | CD194(CCR4) | 2G12 | 100 | Biolegend | 131202 | intracellular |
| 18 | 155Gd | CD127(IL-7Rα) | A7R34 | 100 | Biolegend | 135002 | / |
| 19 | 156Gd | FOXP3 | FJK-16s | 50 | eB | 14-5773-82 | / |
| 20 | 157Gd | TIGIT(VSTM3) | 2190A | 50 | RD | MAB72671 | intracellular |
| 21 | 158Gd | CD45R(B220) | RA3-6B2 | 200 | Biolegend | 103202 | / |
| 22 | 159Tb | F4/80 | Cl:A3-1 | 100 | Biorad | MCA497G | / |
| 23 | 160Gd | TCR β chain | H57-597 | 400 | Biolegend | 109202 | / |
| 24 | 161Dy | CD274(PD-L1) | 10F.9G2 | 100 | Biolegend | 124302 | / |
| 25 | 162Dy | CD103 | 2E7 | 50 | Biolegend | 121402 | / |
| 26 | 163Dy | CD25 | 3C7 | 50 | Biolegend | 101902 | / |
| 27 | 164Dy | CD11c | N418 | 100 | Biolegend | 117302 | / |
| 28 | 165Ho | CD95(Fas) | SA367H8 | 100 | Biolegend | 152602 | / |
| 29 | 166Er | CD192(CCR2) | 475301 | 100 | RD | MAB55381-100 | / |
| 30 | 167Er | CD49b(pan-NK cells) | DX5 | 400 | Biolegend | 108902 | / |
| 31 | 168Er | CD64(FcγRI) | X54-5/7.1 | 50 | Biolegend | 139302 | / |
| 32 | 169Tm | CD279(PD-1) | 29F.1A12 | 200 | Biolegend | 135202 | / |
| 33 | 170Er | MERTK(Mer) | 2B10C42 | 100 | Biolegend | 151502 | / |
| 34 | 171Yb | CD69 | H1.2F3 | 50 | Biolegend | 104502 | / |
| 35 | 172Yb | CD86 | GL-1 | 400 | Biolegend | 105002 | / |
| 36 | 173Yb | Granzyme B | QA16A02 | 50 | Biolegend | 372202 | / |
| 37 | 174Yb | CD27 | LG.3A10 | 50 | Biolegend | 124202 | intracellular |
| 38 | 175Lu | CD196(CCR6) | 29-2L17 | 100 | Biolegend | 129802 | / |
| 39 | 176Yb | CD366(Tim-3) | RMT3-23 | 200 | Biolegend | 119702 | / |
| 40 | 197Au | CD4 | RM4-5 | 800 | Biolegend | 100576 | / |
| 41 | 198Pt | CD8a | 53-6.7 | 400 | Biolegend | 100746 | / |
| 42 | 209Bi | CD11b | M1/70 | 400 | Biolegend | 101202 | / |
